# Effect of bloodstream infection on survival in COVID-19 patients admitted to an intensive care unit in Colombia: a matched cohort analysis

**DOI:** 10.1016/j.infpip.2023.100283

**Published:** 2023-04-29

**Authors:** Jorge Alberto Cortes, Martha Carolina Valderrama-Rios, Laura Cristina Nocua-Báez, Lina María Quitián, Fabio Alexander Lozada, Giancarlo Buitrago

**Affiliations:** a Department of Internal Medicine, Universidad Nacional de Colombia, Sede Bogotá, Facultad de Medicina, Bogotá, Colombia; b Infectious Diseases Service, Hospital Universitario Nacional, Bogotá, Colombia; c Research Institute, Universidad Nacional de Colombia, Sede Bogotá, Facultad de Medicina, Bogotá, Colombia

**Keywords:** COVID-19, SARS-Cov-2, Critical care, Bacteraemia, Mechanical ventilation, Mortality

## Abstract

**Aim:**

To determine the impact of bloodstream infection (BSI) and other risk factors for mortality in patients with COVID-19 admitted to the intensive care unit (ICU).

**Methods:**

A retrospective cohort was carried out at the Hospital Universitario Nacional (HUN) between March 29 and December 19, 2020. Patients with COVID-19 admitted to the Intensive Care Unit (ICU) were paired 1:4 in two groups, one with BSI and the other without, according to hospital stay and the month of admission. The primary outcome was mortality at 28 days. A Cox proportional hazards model was used to estimate differences in mortality risk.

**Results:**

456 patients were identified and 320 were included in the final cohort, 18% (n = 59) in the BSI group and 82% (n = 261) in the control group. 125 (39%) patients died, 30 (51%) in the BSI group and 95 (36%) in the control group (*P* = 0.040). BSI was associated with an increased risk of in-hospital mortality at 28 days, [HR] 1.77 (95% CI: 1.03–3.02; *P* = 0.037). Invasive mechanical ventilation (IMV) and age were associated with increased mortality risk. Some months of the year of the hospital stay were associated with a reduced risk of mortality. There was no difference in mortality between inappropriate and appropriate empirical antimicrobial use.

**Conclusion:**

BSI in patients with COVID-19 in ICU increases in-hospital mortality to 28 days. Other risk factors for mortality were IMV and age.

## Introduction

Various complications have been identified among COVID-19 patients that can lead to death. According to retrospective studies conducted in Italy and China, mortality rates have been reported to range from 44.3% to 61.5% [1,2] in patients with severe disease due to COVID-19 who require hospitalisation in an intensive care unit (ICU) in the first half of 2020. The complications found include acute respiratory distress syndrome (ARDS), pulmonary thromboembolism, and secondary infections such as pneumonia and bloodstream infections (BSI) [3], which can be very difficult to differentiate clinically and which can occur concomitantly.

The frequency of BSI in patients with COVID-19 infection who are admitted to the ICU is higher than in those without COVID-19, as reported in a cohort study published by Cataldo *et al.*, in which it was identified that the prevalence of BSI in the period pre-COVID-19 was 3.8 times lower [4]. A study by Cuntro *et al.* identified that the microorganisms most frequently identified in blood cultures from patients with BSI and SARS-CoV-2 infection were Enterobacterales and Gram-positive cocci [5]. The clinical presentation resulting from sepsis of bacterial or fungal aetiology is, in many cases, difficult to distinguish from sepsis due to COVID-19, which has increased the indiscriminate use of antimicrobials in patients with severe disease caused by SARS-CoV-2 [6].

The impact of these BSI on patients with severe COVID-19 in the ICU is not clear. In order to determine the impact of this complication on in-hospital mortality in patients with SARS-CoV-2 infection admitted to the ICU, the present retrospective cohort study was carried out.

## Methods

### Setting and inclusion criteria

This study was conducted at the Hospital Universitario Nacional (HUN), a highly complex referral center in Bogotá, Colombia. The patients referred to the institution come from Bogotá and other cities in Colombia and belong to various types of insurance. Patients could enter the ICU directly or be admitted to the floor and, due to clinical deterioration, be admitted to the ICU. The patients included were adults aged 18 years or older, with a confirmed SARS-CoV-2 infection diagnosis by RT-PCR test or a suspected diagnosis defined by the treating medical group. They required in-hospital treatment from March 29, 2020, to December 19, 2020, and were followed up from the hospital admission date to discharge or death, whichever occurred first. Patients with missing data due to non-registration in the electronic medical record or transfer to another institution were excluded, as well as those in whom blood culture contamination was considered (see below). The research ethics committee of the Hospital Universitario Nacional de Colombia, Bogotá, approved the study, confirming that patient consent was not necessary for this low risk study (CEI-HUN-ACTA-2020-03).

### Microbiological data and exposure

Based on the identification of BSI during follow-up, we defined 2 mutually exclusive exposure groups. Exposure group, defined as patients with BSI, and non-exposed group, defined as patients without BSI. BSI was defined as the isolation of bacteri or fungi from blood cultures using Bactec FX systems (Beckton Dickinson, NJ, USA). The treating medical team requested blood cultures according to the patient's clinical condition, and blood cultures were collected according to the parameters defined by local regulatory entities [7]. Two sets of aerobic blood cultures (consisting of two bottles) were taken, and occasionally, a third set of anaerobic or fungal blood cultures (comprising of two bottles). Once the blood cultures were positive, identification and susceptibility were performed using the automated system BD Phoenix (Beckton Dickinson, NJ, USA). When *Candida auris* was isolated from blood cultures, the isolate was sent to the National Institute of Health for confirmation.

Each patient was included and analysed only once. The data corresponding to the first microbiological isolation identified by blood culture was included in patients in the exposure group. Patients with contaminated blood cultures, defined in this study as the microbiological isolation of coagulase-negative *Staphylococcus spp.* in only 1 out of 2 blood cultures, were excluded.

In the patients with BSI, the empirical antimicrobial treatment was classified into 2 groups as appropriate or inappropriate. Appropriate antimicrobial treatment was defined if the microorganism grown was found to be susceptible *in vitro* to the empirical antimicrobial treatment. Additionally, as the HUN is a referral centre we classified the BSI as: Group 1. Acquired in a hospital other than ours (another hospital), identified within 48 hours of arrival; Group 2. Acquired in our hospital (this hospital), identified within 48 hours of arrival.

### Outcomes and covariates

The primary outcome was in-hospital mortality. The following sociodemographic data and clinical characteristics were considered as possible confounding factors and variables to be measured: age, sex, Charlson comorbidities index, body mass index (BMI), the relationship between arterial oxygen pressure and inspired fraction of oxygen on admission (PaO2/FiO2), and the need for invasive mechanical ventilation (IMV).

The variables were evaluated using the retrospective data available in the hospital's electronic medical record. The variables of IMV and in-hospital mortality were evaluated from the date of admission to discharge or death, whichever occurred first. IMV was considered when it preceded BSI at least 24 hours before the date on which the blood cultures were sampled. Patients with missing data for more than two covariates were excluded due to not recording the electronic medical data or due to transfer to another institution.

### Statistical analysis

A retrospective analysis of matched cohorts was performed. To equalise the risk of BSI by the length of stay in ICU, patients with BSI were identified during follow-up and were paired 1:4 with patients without it who were hospitalised during the same time.

A Cox proportional hazards model was used to estimate differences in mortality risk between the two cohorts as a risk ratio and to model the probability of survival over time. The covariate or confounding factors that were included to adjust the estimates of mortality risk were: sex, age, Charlson comorbidity index, body mass index (BMI), PaO2/FiO2 ratio, the need for IMV, the days of hospital stay, and the month of the year of exposure. Analyses were performed using STATA software (STATA 17, StataCorp, TX, USA).

## Results

**Figure 1:**
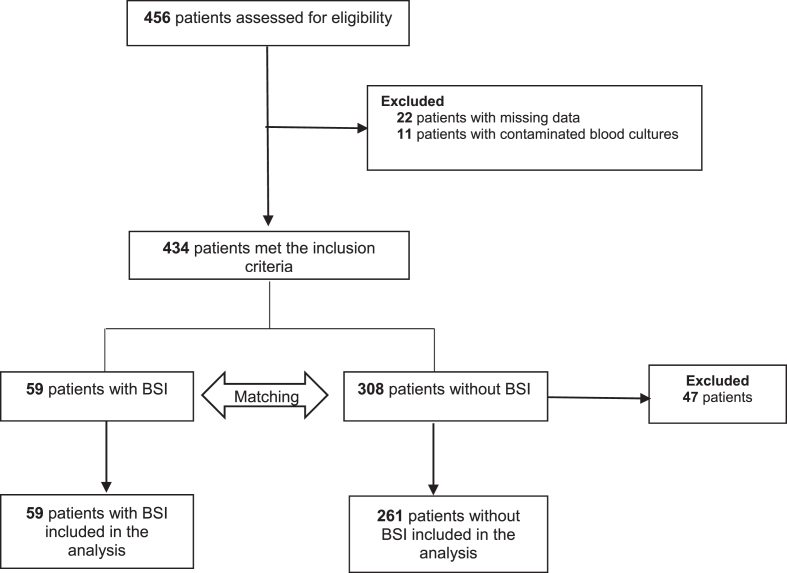
Flow chart of the patients included in the study.

456 adult patients were identified in the HUN with confirmed or suspected SARS-CoV-2 infection, with an indication for in-hospital management, from March 29, 2020, to December 19, 2020. After applying the inclusion/exclusion criteria, 78 patients were excluded for missing data and 11 patients for contaminated blood cultures. After pairing, 47 patients from the cohort without BSI were excluded for no match. The total cohort consisted of 320 patients, 18% (n = 59 patients) in the group of patients with BSI and 82% (n = 261 patients) in the control cohort (Figure 1). Of the 59 patients with BSI, we determined that 23.8% (14/59) were Group 1, acquired in another hospital; 37.3% (22/59) were Group 2, acquired in our hospital and in 38.9% (23/59) cases this information was not available. In the total cohort, the median age was 62 years (IQR 51–72 years), the majority of patients were men (69%), were overweight or obese (73%), and had a severe respiratory failure at the time of admission (44%) and required IMV during hospital stay (79%). The characteristics of the patients for each of the two cohorts (BSI/no BSI) are shown in Table I.

**Table I:** Patient baseline characteristics

Characteristic	Total n=320	BSI n = 59	No BSI n = 261
Men, n (%)	221 (69)	40 (68)	181 (69)
Age, n (%)			
≤ 50 years	74 (23)	10 (17)	64 (24)
51–60 years	56 (18)	7 (12)	49 (19)
61–70 years	88 (28)	16 (27)	72 (28)
71–80 years	80 (25)	17 (29)	63 (24)
≥ 81 years	27 (6)	9 (15)	13 (5)
Charlson, median (IQR)	2.5 (1–4)	3 (2–4)	2 (1–4)
Cardiac insufficiency	38 (12)	7 (11)	31 (12)
Chronic obstructive pulmonary disease	38 (12)	12 (20)	26 (10)
Diabetes mellitus	89 (28)	19 (33)	70 (27)
Neoplasia	22 (7)	4 (7)	18 (7)
BMI, n (%)			
<18.5	2 (1)	0	2 (1)
≥ 18.5–24.9	85 (26)	10 (17)	75 (29)
25.0–29.9	100 (31)	21 (36)	79 (30)
≥ 30	133 (42)	28 (47)	105 (40)
PaO2/FiO2, n (%)			
≤ 100 mmHg	140 (44)	29 (49)	111 (43)
101–200 mmHg	105 (33)	22 (37)	83 (32)
201–300 mmHg	60 (19)	7 (12)	53 (20)
> 301 mmHg	15 (4)	1 (2)	14 (5)
Invasive mechanical ventilation, n (%)	254 (79)	53 (90)	201 (77)
Death 28 days after hospital admission	125 (39)	30 (51)	95 (36)

### Pattern of microorganisms isolated

In the 59 BSI patients, 65 microorganisms were identified, including six mixed infection BSI cases. The most frequently isolated pathogens were Gram-negative bacilli in 52 cases (89%), Gram-positive cocci in 7 cases (12%), and 6 yeasts (10%). The microorganisms identified are shown in Table II. The resistance phenotype identified was typical in 33 cases (56%), and production of beta-lactamases 23 isolates of Enterobacterales (53% of isolates of this type) was phenotypically identified, including AmpC in 9 isolates (15% of patients), extended-spectrum beta-lactamases in 2 cases and 3 isolates of carbapenemase-producing *K. pneumoniae* (18% of the isolates of this species). In total, 4 patients had a carbapenemase-producing organism isolated (7% of patients). Table III shows the resistance profile of Gram-negative microorganisms.

**Table II:** Microorganisms identified in BSI

Microorganism	Number (%)
Gram-negative bacilli	52 (86)
*Klebsiella pneumoniae*	17 (29)
*Escherichia coli*	9 (15)
*Pseudomonas aeruginosa*	6 (10)
*Klebsiella oxytoca*	6 (10)
*Enterobacter cloacae*	5 (8)
Other Gram-negative bacilli *Proteus mirabilis* *Klebsiella aerogenes* *Citrobacter freundii* *Pseudomonas putida* *Pantoea agglomerans* *Burkholderia cepacia* *Acinetobacter baumannii complex*	9 (15) 3 1 1 1 1 1 1
Gram-positive cocci	7 (12)
*Enterococcus faecalis*	3 (5)
*Staphylococcus aureus*	2 (3)
*Enterococcus faecium*	1 (2)
*Streptococcus mitis*	1 (2)
Yeasts	6 (10)
*Candida auris*	2 (3)
*Candida albicans*	2 (3)
*Candida parapsilopsis*	2 (3)
Mixed infections Bacteria + yeast combination Combination of 2 different bacteria	6 (10) 3 3

**Table 3:** Resistance profile of Gram-negative microorganisms

Microorganism (N total)	%	Penicillin	Piperacillin/Tazobactam	3rd generation Cephalosporin	Carbapenem	Ciprofloxacin	Gentamicin
***Klebsiella pneumoniae (17)***	**100**	**X**^a^					
	**29.4**		**X**				
	**23.5**			**X**			
	**17.6**				**X**		
	**11.8**					**X**	
***Klebsiella oxytoca* (6)**	**100**	**X**^a^					
	**16.7**		**X**				
	**16.7**			**X**			
***Echerichia coli (9)***	**66.7**	**X**					
	**22.2**		**X**				
	**11.1**			**X**			
***Pseudomonas aeruginosa (6)***	**16.7**		**X**				
	**66.7**			**X**			
	**16.7**				**X**		
	**50**					**X**	
***Enterobacter spp. (6)***	**100**	**X**^a^					
	**100**			**X**^a^			
***Proteus mirabilis (3)***	**66.6**	**X**					
	**33.3**		**X**				
	**33.3**				**X**		
***Citrobacter freundii (1)***	**100**			**X**			

There was one isolate of Pseudomonas putida and of Pantoea agglomerans that did not show resistance. We found an isolate of Burkholderia cepacia and Acinetobacter baumannii which presented a typical susceptibility profiles, with inherent resistance to ampicillin and first-generation cephalosporins.

a Inherent resistance to ampicillin.

In the empirical antimicrobial treatment for the BSI patients, treatment was appropriate in 42 cases (71%), with no empirical antifungal treatment observed in any of the cases of candidaemia. In-hospital mortality at 28 days in the group of patients with appropriate empirical antimicrobial treatment was 50% (n = 21 patients), compared with 59% (n = 10 patients) in the group of patients with inappropriate treatment (*P* = 0.539).

### Impact of risk factors on mortality

**Figure 2:**
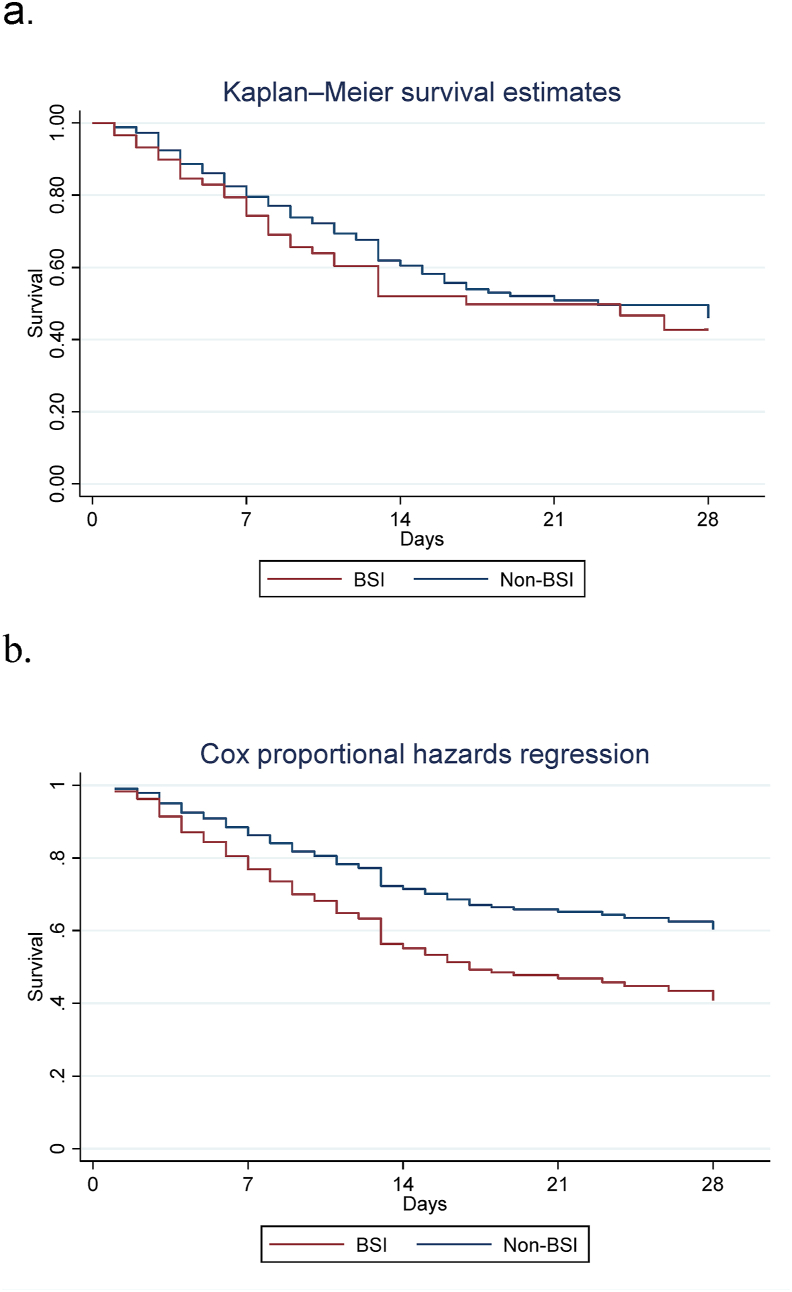
Survival curves evaluating the effect of BSI in patients with COVID-19 in ICU. Survival curves at 28 days evaluating the effect of BSI. Panel 2a is unadjusted, and panel 2b with subsequent adjustment to Cox proportional hazards models.

In the total cohort of 125 patients (39%), 30 patients (51%) in the BSI group, and 95 patients (36%) in the no BSI cohort died (*P*= 0.040). The Kaplan-Meier analysis did not reveal a statistically significant difference in time to mortality among BSI patients compared to patients without BSI (log-rank test *P* = 0.420) (Figure 2a). When adjusting the results in a Cox proportional risk model with other covariates (sex, age, Charlson comorbidity index, BMI, PaO2/FiO2 ratio, need for IMV, days of hospital stay, and month of the year of exposure), the BSI sample was associated with an increased risk of in-hospital mortality at 28 days, hazard ratio [HR] 1.77 (95% CI: 1.03–3.02; *P* = 0.037) (Figure 2b). In the Cox proportional hazards model, invasive mechanical ventilation was also associated with a statistically significant increased risk of mortality, hazard ratio [HR] of 3.56 (95% CI: 1.81–6.99; *P* < 0.001). In contrast, the month of the hospital stay was associated with a reduction in mortality risk, reaching 78% in October (Table IV).

**Table IV:** Cox proportional risks model for mortality

	HR	95% CI	*P*-value
BSI	1.77	1.03–3.02	0.037
Gender: Male	1.05	0.72–1.53	0.772
Age	1.04	1.01–1.08	0.002
Charlson ≥ 5	0.77	0.20–2.99	0.711
BMI			
Low	1.84	0.16–20.34	0.616
Normal	1.24	0.84–1.82	0.273
Obesity	0.88	0.55–1.41	0.600
PaO2/FiO2	0.99	0.99–1.00	0.058
Invasive mechanical ventilation	3.56	1.81–6.99	<0.001
Days of hospital stay	0.92	0.88–0.97	0.003
Month of hospitalisation			
June	0.31	0.15–0.62	0.001
July	0.47	0.21–1.06	0.070
August	0.27	0.13–0.57	<0.001
September	0.45	0.21–0.95	0.037
October	0.22	0.09–0.52	0.001
November	0.52	0.23–1.19	0.124
December	0.49	0.22–1.08	0.077

## Discussion

This study of COVID-19 patients admitted to the ICU in a referral hospital in Colombia found that BSI was a risk factor that increased the mortality of these critically ill patients after adjusting for other variables.

Our findings are consistent with those described in Italy, where more deaths were reported in patients with BSI and COVID-19 (38.6%) compared with those patients who did not present this coinfection (24.2%) [8] and findings described in France, which reported a higher mortality rate in patients with BSI and COVID-19, compared to those who did not present BSI (39% vs. 33%, *P*=0.036), in an analysis with a propensity score. The researchers also found that the diagnosis of BSI represented 3.6% of the general mortality of the population and that patients with this condition died faster vs. individuals without both infectious diseases (HR 1.28, 95% CI 1.05–1.56) [9]. A Swedish study reported an increased mortality risk at 30 days in BSI and COVID-19 patients compared to those who had BSI without COVID-19 (adjusted OR 2.44, 95% CI 1.75–3.38) [10]. The early onset of bacterial or fungal BSI has been reported to be independently related to a higher rate of fatal outcomes (HR 4.68, 95% CI 1.40–15.63) [11].

The mortality rate from COVID-19 varies depending on the hospital scenario, complications, attention span, and circulating virus. In studies carried out in Wuhan, China, a mortality of 16.6% was documented in intensive care hospitalised patients [12]. A rate of 30.9% was observed in critically ill patients during the first wave of COVID-19 in the United States [13], while additional information from 2020, with data from 5700 patients with COVID-19, it was found that up to 14.3% of patients required ICU stay, with a mortality of 24.5% related to the requirement for invasive mechanical ventilation [14]. A systematic review and meta-analysis of the first year of the pandemic identified 46 studies in which it found a higher incidence of BSI in patients with COVID-19 than without it (OR 2.77; 95% CI 1.53–5.02), with an incidence of 29.6% of patients admitted to the ICU (95% CI, 21.7–31.8%) and with a mortality of 41% (95% CI, 30–52.8%), a range within which our data are found. The data also seem consistent with those observed in the first and second waves in Colombia, with mortality that seemed to be affected by the time at which patients were treated [15], similar to that observed in other Latin American countries [16].

Lymphopaenia, endothelial dysfunction, and changes in micro- and macrocirculation that predispose to intestinal bacterial translocation have been observed in patients with SARS-CoV-2 infection. These factors seem to favour bacterial superinfection, which may be associated with the development of BSI [17,18]. The pathophysiological involvement of the gastrointestinal tract is part of the argument explaining why the microorganisms most frequently isolated in BSI in patients with COVID-19 are Gram-negative, as in our study [19]. Previously, it has been reported that in patients with respiratory virus infections such as influenza [20]. There is a dysregulation of the immune system at the epithelial level and, in general, of the immune response mediated by T and B lymphocytes, which predisposes to secondary bacterial infection [20]. In a systemic review, it was found that 1 out of 4 patients with H1N1 infection in ICU had secondary bacteraemia, which implied a higher risk of mortality [21].

One of the protective factors is related to the month of the year in which COVID-19 patients were hospitalised. This variable was introduced in the model to recognise the changes that were made in the care of patients during the first wave in Colombia, which may be related to the workload, as has been described in Israel, where a higher mortality rate was reported in the periods of the year with a higher workload [22]. During the COVID-19 pandemic peaks in ICUs, the activities of health personnel as nurses have been considerably greater compared with non-COVID-19 time [23]. Other factors that may be involved in the mortality rate include the use of medications such as steroids.

Our study has some limitations. First, it was carried out in a single healthcare centre, which, because it was a referral centre for patients with SARS-CoV-2 infection, could mean that the patients included had more severe illness. Second, it was impossible to determine whether the bacteraemia events may have represented healthcare-associated infection and whether the implementation of preventive strategies to reduce healthcare-associated BSI, could have reduced the mortality rate of patients with SARS-CoV2 infection in ICU. On the other hand, one of the strengths of this study is the matched pairing as a strategy for the control of possible confounding variables, precisely the time of stay in the ICU, since the characteristics of some of the risk factors such as BSI and IMV, means that it is not possible to carry out investigations through clinical trials.

In conclusion, in adult patients with COVID-19 in the ICU, the presence of a BSI increases the risk of in-hospital mortality at 28 days. Other identified risk factors include IMV and age.

## Conflict of interests

J.A.C. and M.C.VR have a grant from the International Infectious Diseases Society supported by Pfizer. L.C.NB, L.M.Q, F.A.L., and G.B. have no conflict of interest to declare.

## Funding

No specific funding was allocated for the present study.

## Access to data

The datasets from the study are available from the corresponding author on request.

## Contributions

J.A.C. conceived, coordinated the data collection and supervised the study. J.A.C. and G.B designed the sdtudy. L.C.NB, L.M.Q, and F.A.L. collected and interpreted the data. M.C.VR and G.B. performed the statistical analysis. J.A.C. and L. CNB. wrote the first draft of the manuscript. All authors revised the manuscript and approved the final version of the manuscript.
